# The Good pH probe: non-invasive pH in-line monitoring using Good buffers and Raman spectroscopy

**DOI:** 10.1007/s00216-023-04993-0

**Published:** 2023-11-20

**Authors:** David Heinrich Müller, Marieke Börger, Julia Thien, Hans-Jürgen Koß

**Affiliations:** Institute of Technical Thermodynamics, RWTH Aachen University, Schinkelstraße 8, 52062 Aachen, Germany

**Keywords:** Raman spectroscopy, pH value, In-line monitoring, Good buffers, 3-(*N*-Morpholino)propanesulfonic acid (MOPS), Indirect Hard Modeling (IHM)

## Abstract

In bioprocesses, the pH value is a critical process parameter that requires monitoring and control. For pH monitoring, potentiometric methods such as pH electrodes are state of the art. However, they are invasive and show measurement value drift. Spectroscopic pH monitoring is a non-invasive alternative to potentiometric methods avoiding this measurement value drift. In this study, we developed the Good pH probe, which is an approach for spectroscopic pH monitoring in bioprocesses with an effective working range between pH 6 and pH 8 that does not require the estimation of activity coefficients. The Good pH probe combines for the first time the Good buffer 3-(*N*-morpholino)propanesulfonic acid (MOPS) as pH indicator with Raman spectroscopy as spectroscopic technique, and Indirect Hard Modeling (IHM) for the spectral evaluation. During a detailed characterization, we proved that the Good pH probe is reversible, exhibits no temperature dependence between 15 and 40 °C, has low sensitivity to the ionic strength up to 1100 mM, and is applicable in more complex systems, in which other components significantly superimpose the spectral features of MOPS. Finally, the Good pH probe was successfully used for non-invasive pH in-line monitoring during an industrially relevant enzyme-catalyzed reaction with a root mean square error of prediction (RMSEP) of 0.04 pH levels. Thus, the Good pH probe extends the list of critical process parameters monitorable using Raman spectroscopy and IHM by the pH value.

**Graphical abstract:**

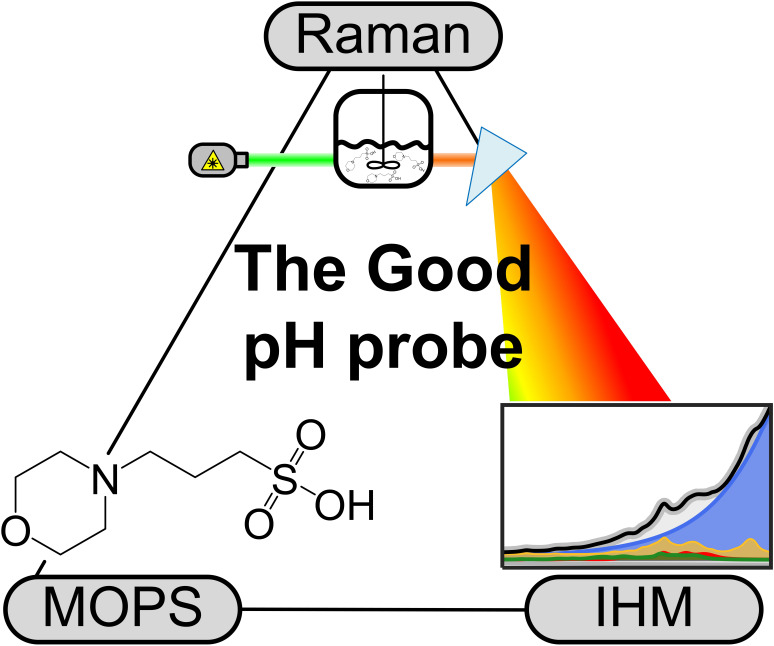

## Introduction

In bioprocesses, the pH value is a crucial operating parameter [1]. A pH optimum is given for both enzymes and microorganisms, at which they work most productively [2–4]. The pH value is the negative common logarithm of the hydronium ion activity ${a}_{{\mathrm{H}}_{3}{\mathrm{O}}^{+}}$:

1 $$\mathrm{pH}=-{\mathrm{log}}_{10}\left({a}_{{\mathrm{H}}_{3}{\mathrm{O}}^{+}}\right).$$

To monitor the pH value, potentiometric methods such as pH electrodes and ion-sensitive field-effect transistors (ISFETs) are the state of the art [5]. However, classical pH electrodes show several disadvantages. First, pH electrodes are invasive, since they must be immersed into the monitored system. Second, pH electrodes show measurement value drift, resulting in the need for frequent recalibration or, in the case of bioreactors, pH off-line measurements for verification [6–10]. Third, pH electrodes cannot be applied in small-scale systems such as mini-bioreactors [11]. Unlike pH electrodes, ISFETs are applicable in mini-bioreactors, but still show measurement value drift [12].

An alternative to potentiometric methods for pH monitoring is the use of spectroscopic techniques [13]. These techniques are potentially non-invasive, are not expected to show measurement value drift, and are suitable for the application in mini-bioreactors [13–15]. To determine the pH value, spectroscopic techniques must be combined with a pH indicator, which is usually a weak acid or base that protonates or deprotonates as a function of the pH value [6, 13]. Spectroscopic techniques can be used to determine the concentrations of the protonated and the deprotonated pH indicator species, which are then typically correlated with the pH value [6, 13, 16].

If the pH indicator is not already part of the investigated system and must be integrated additionally, interactions with other components may occur. Therefore, pH indicators are often immobilized in a solid matrix to minimize indicator leaching into the investigated system [6, 13]. An immobilized pH indicator can be integrated into a system as sensor spot or coating [6, 13, 17]. State-of-the-art immobilization approaches can nearly completely prevent pH indicator leaching [13]. However, even an immobilized pH indicator can perturb the investigated system if the pH indicator acts as a pH buffer [13]. This effect is referred to as indicator error and is especially relevant in small-scale applications such as mini-bioreactors [13].

If the pH indicator is already part of the system, leaching effects, additional immobilization effort, and the indicator error can be excluded. In most bioprocesses, pH buffers are applied to compensate minor pH changes [18, 19]. Usually, pH buffers are weak acids or bases just like pH indicators [13, 18, 20]. Therefore, the use of pH buffers as pH indicators in bioprocesses enables completely non-invasive pH monitoring [20–24].

To use pH buffers as pH indicators, these buffers must be combined with an appropriate spectroscopic technique. Raman spectroscopy is a widely used process analytical technology (PAT) tool in bioprocesses, but its application for pH monitoring remains to be extensively explored [13, 25]. It is a spectroscopic technique that evaluates the inelastic light scattering [26]. Raman spectroscopy shows high specificity and can provide quantitative information about the sample composition [27]. The latter is due to the fact that the Raman scattering intensity ${I}_{\mathrm{Raman},i}$ of a component $i$ correlates linearly with the component’s number of molecules ${N}_{i}$ in the measurement volume and therefore also with its concentration ${c}_{i}$ [26, 28]:

2 $${I}_{\mathrm{Raman},i}\propto {N}_{i}\propto {c}_{i}.$$

Thus, Raman spectroscopy cannot only be combined with a pH buffer for pH monitoring, but can also be used for simultaneous monitoring of multiple process parameters of a bioprocess such as substrate, metabolite, and product concentrations.

A common challenge for spectroscopic pH monitoring is its sensitivity to the ionic strength [13]. In contrast to potentiometric methods, which provide information on the hydronium ion activity, spectroscopic techniques can only measure concentration-based information, as stated above [6]. The hydronium ion activity ${a}_{{\mathrm{H}}_{3}{\mathrm{O}}^{+}}$ is defined as

3 $${a}_{{\mathrm{H}}_{3}{\mathrm{O}}^{+}}=\frac{{c}_{{\mathrm{H}}_{3}{\mathrm{O}}^{+}}}{{c}_{0}}\cdot {\gamma }_{{\mathrm{H}}_{3}{\mathrm{O}}^{+}},$$

where ${c}_{{\mathrm{H}}_{3}{\mathrm{O}}^{+}}$ is the hydronium ion concentration, ${c}_{0}$ the standard concentration (1 mol/L), and ${\gamma }_{{\mathrm{H}}_{3}{\mathrm{O}}^{+}}$ the hydronium ion activity coefficient [29]. Activity coefficients are a function of the ionic strength [6, 13]. Therefore, spectroscopic pH monitoring is sensitive to this parameter and is limited in its accuracy if activity coefficients are not estimated [6, 13].

Metcalfe et al. [22] used Raman spectroscopy as spectroscopic technique and combined it with phosphate buffer as pH indicator for non-invasive on-line monitoring of the pH value during a mixed acid fermentation of *Escherichia coli* (*E. coli*). They were able to monitor the pH value between pH 6 and pH 8 with an accuracy of better than 0.1 pH levels. To address the sensitivity of phosphate buffer to the ionic strength and improve the prediction quality, Metcalfe et al. [22] estimated the activity coefficients for their experiments. In their estimation, the activity coefficients were computed as a function of the ionic strength [22–24]. The ionic strength can be determined either by calculation, which may be effortful and requires much process knowledge, or by additional measurement techniques such as conductometry [13].

In contrast to phosphate buffer, the Good buffer 3-(*N*-morpholino)propanesulfonic acid (MOPS) has only one degree of dissociation, making it less sensitive to the ionic strength than pH indicators with higher degrees of dissociation [13, 24]. The frequently used Good buffers were specifically developed for application in bioprocesses by Good et al. [30] and have many advantages in this field. These advantages include effective buffering in the physiological range between pH 6 and pH 8, no participation in biological reactions, low biological membrane permeability, low toxicity, high water solubility, and high resistance to both enzymatic and non-enzymatic degradation [30–32]. Further advantages are little influences of the temperature, buffer concentration, and ionic strength on the dissociation of the buffer [30, 32].

In this study, we show that the Good buffer MOPS can be used as pH indicator for non-invasive pH in-line monitoring in bioprocesses without requiring the estimation of activity coefficients. For this purpose, we combined MOPS as pH indicator with Raman spectroscopy as spectroscopic technique and with Indirect Hard Modeling (IHM) as spectral evaluation model. Since IHM is physics-based, it can provide robust models for evaluating bioprocess Raman spectra with significantly reduced calibration effort compared with state-of-the-art statistical models [33, 34].

We call this approach “the Good pH probe”. To the best of our knowledge, this is the first application of a Good buffer as pH indicator, and the first use of IHM to evaluate Raman spectra for spectroscopic pH monitoring. With respect to our previous study, in which we in-line monitored the substrate and product mass fractions in a bioprocess by combining Raman spectroscopy with IHM, the Good pH probe adds the pH value to the list of critical process parameters that can be monitored using this combination [34]. We thoroughly characterized the Good pH probe in regard to its reversibility, temperature dependence, sensitivity to ionic strength, and applicability in systems with components whose Raman spectra strongly superimpose the spectral features of MOPS. Finally, to demonstrate the Good pH probe’s applicability in bioprocesses, we used it to non-invasively in-line monitore the pH value during the industrially relevant enzyme-catalyzed hydrolysis of penicillin G to 6-aminopenicillanic acid and phenylacetic acid by penicillin amidase.

## Materials and methods

### Materials and reagents

Water (SupraSolv^®^, Supelco^®^) and 30 wt% hydrochloric acid (HCl) solution (Suprapur^®^, Supelco^®^) were purchased from Merck KGaA. Sodium hydroxide (NaOH) solution (38.5 wt%) was prepared from NaOH platelets (EMSURE^®^, Supelco^®^) acquired from Merck KGaA. MOPS (Ultrapure) and sodium chloride (NaCl) were obtained from VWR. Penicillin amidase from *E. coli* with enzyme activity of 711 U/mL was purchased from Sigma-Aldrich Chemie GmbH, and penicillin G potassium salt (CELLPURE^®^) from Carl Roth GmbH & Co. KG.

### Measurement setup

**Fig. 1:**
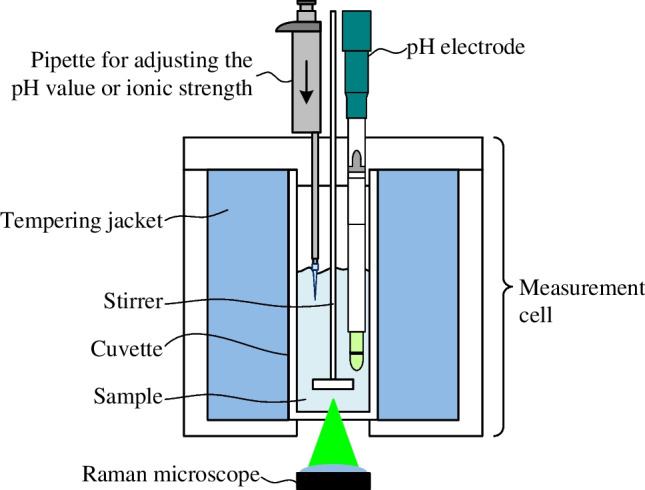
Schematic representation of the measurement setup. The setup consists of the following components: measurement cell, Raman microscope, pH electrode, and a pipette to add solutions for adjusting the pH value or the ionic strength of the sample. The measurement cell includes the cuvette, the tempering jacket, and the stirrer

To enable the use of a reference pH electrode, all experiments were performed in a customized measurement cell, which is part of the measurement setup shown in Fig. 1. The customized measurement cell includes a glass cuvette with an inner diameter of 30 mm, a height of 75 mm, and a volume of 53 mL. The glass cuvette is surrounded by a tempering jacket. Through this tempering jacket, water from an external thermostat (CC-K15 with Pilot ONE; Huber) was cycled to achieve temperature control. The bottom of the cuvette is flat and accessible by the laser of an inverse confocal Raman microscope (InVia; Renishaw). We used a laser with a wavelength of 532 nm and a laser power of 100 mW in combination with an 1800 l/mm grating. The laser light was focused in the sample with a microscope objective (LmPlanFL N, 20x, NA = 0.4; Olympus).

For pH reference measurements, the top of the measurement cell enables the integration of a pH electrode (InLab Micro Pro-ISM; Mettler-Toledo), which is coupled to a pH meter (SevenCompact S210; Mettler-Toledo). The pH electrode was calibrated prior to each experiment, and the manufacturer reported a measurement error of approximately ± 0.05 pH levels. Moreover, the pH electrode contains a temperature sensor that was used for temperature compensation by the pH meter as well as for temperature monitoring of the sample in the cuvette. Solutions for adjusting the pH value or the ionic strength in the sample were added through an aperture in the top of the measurement cell using a pipette (Transferpette^®^ S; Brand). After these additions, a built-in motorized stirrer was used to quickly homogenize the sample inside the cuvette.

### Experimental procedure

Our experimental procedure encompassed the experiments regarding the Good pH probe’s calibration, its characterization, and an experiment to prove its applicability in bioprocesses. For the calibration and characterization experiments, we followed the experimental procedure of a standard experiment and modified it with respect to parameters such as temperature or sample composition. The specific modifications made are described in the “Calibration of the Good pH probe” and “Characterization of the Good pH probe” sections.

To perform a standard experiment, we prepared 30 mL of an aqueous sample containing 50 mM MOPS, placed it inside the cuvette of the measurement cell, and tempered it. Next, we repeatedly added a solution to the sample to gradually change either its pH value or ionic strength. After each addition, we first stirred the sample for 1 min and then waited for another minute before we recorded the reference pH value and the Raman spectra.

For the Good pH probe’s application experiment, the pH in-line monitoring during an enzyme-catalyzed reaction, we prepared an aqueous solution containing 50 mM MOPS and 50 mM penicillin G. This solution was tempered to 25 °C and its pH value was adjusted to approximately pH 8.2 by adding NaOH solution. The enzyme-catalyzed reaction was initiated by introducing penicillin amidase from *E. coli*, leading to an enzyme activity of 0.44 U/mL in the sample. Throughout the reaction, the reference pH value was monitored each minute and Raman spectra were recorded continuously. In contrast to the calibration and characterization experiments, during the Good pH probe’s application experiment, no solutions were added to the sample and the sample was not stirred.

### Raman spectra

For all experiments, the Good pH probe’s Raman spectra were recorded as single point acquisitions in the spectral range from 2505 to 3825 cm^−1^. In the calibration and characterization experiments, for every Raman spectrum, 45 acquisitions with an excitation time of 4 s each were recorded and integrated, resulting in an accumulated excitation time of 180 s. To increase the temporal resolution during the enzyme-catalyzed reaction, we recorded and integrated only 15 acquisitions for each Raman spectrum to obtain an accumulated excitation time of 60 s. None of these Raman spectra was pretreated before the spectral evaluation using IHM.

### Calculation of the pH value

The dissociation reaction of MOPS

4 $$\mathrm{MOPSH}+{\mathrm{H}}_{2}\mathrm{O}\rightleftharpoons {\mathrm{MOPS}}^{-}+{\mathrm{H}}_{3}{\mathrm{O}}^{+},$$

where $\mathrm{MOPSH}$ represents the protonated and ${\mathrm{MOPS}}^{-}$ the deprotonated MOPS species, is described by the law of mass action:

5 $${K}_{\mathrm{a},\mathrm{MOPS}}=\frac{{a}_{{\mathrm{MOPS}}^{-}}\cdot {a}_{{\mathrm{H}}_{3}{\mathrm{O}}^{+}}}{{a}_{\mathrm{MOPSH}}}.$$

The thermodynamic acid dissociation constant ${K}_{\mathrm{a},\mathrm{MOPS}}$ includes the water activity, which is commonly assumed to be constant. Using the definition of the pH value [Eq. (1)], the definition of the activity [Eq. (3)], Eq. (5), and the negative common logarithm, the pH value can be calculated as follows:

6 $$\begin{array}{l}\mathrm{pH}=\mathrm{p}{K}_{\mathrm{a},\mathrm{MOPS}}+{\mathrm{log}}_{10}\left(\frac{{a}_{{\mathrm{MOPS}}^{-}}}{{a}_{\mathrm{MOPSH}}}\right)\\ =\mathrm{p}{K}_{\mathrm{a},\mathrm{MOPS}}+{\mathrm{log}}_{10}\left(\frac{{\gamma }_{{\mathrm{MOPS}}^{-}}}{{\gamma }_{\mathrm{MOPSH}}}\right)+{\mathrm{log}}_{10}\left(\frac{{c}_{{\mathrm{MOPS}}^{-}}}{{c}_{\mathrm{MOPSH}}}\right).\end{array}$$

Here, $\mathrm{p}{K}_{\mathrm{a},\mathrm{MOPS}}$ is the negative common logarithm of ${K}_{\mathrm{a},\mathrm{MOPS}}$.

For the prediction of components’ activity coefficients, an empirical extension of the Debye–Hückel theory is often used:

7 $${\mathrm{log}}_{10}\left(\gamma \right)=\frac{-A\cdot {z}^{2}\cdot \sqrt{I}}{1+B\cdot \sqrt{I}}+b\cdot I.$$

In this equation, $A$ is a temperature-dependent parameter, $z$ is the component’s charge, $I$ is the ionic strength, $B$ is a parameter dependent on the component’s ion radius, and $b$ is an empirical parameter that is also specific to the component [20, 29]. According to Eq. (7), the activity coefficient of a component at a given temperature is solely a function of the ionic strength [18, 20, 24, 29]. Furthermore, it can be concluded from Eq. (7) that a pH indicator’s sensitivity to the ionic strength strongly correlates with its charge. As MOPS is either neutrally charged (protonated) or simply negatively charged (deprotonated), it is less sensitive to the ionic strength than more strongly charged pH indicators such as phosphate buffer [13]. Therefore, for the Good pH probe, we assumed the common logarithm of the ratio between ${\gamma }_{{\mathrm{MOPS}}^{-}}$ and ${\gamma }_{\mathrm{MOPSH}}$ to equal 0:

8 $${\mathrm{log}}_{10}\left(\frac{{\gamma }_{{\mathrm{MOPS}}^{-}}}{{\gamma }_{\mathrm{MOPSH}}}\right)=0.$$

This assumption potentially deteriorated the Good pH probe’s prediction quality. However, by investigating the Good pH probe’s sensitivity to the ionic strength, we will finally validate this assumption and show that the prediction error resulting from Eq. (8) is within the range of the inherent measurement error of the reference pH electrode used in our study (“Characterization of the Good pH probe” section). For the Good pH probe, this assumption eliminated the need to estimate activity coefficients as a function of the ionic strength, whose calculation may be effortful or may require additional measurement techniques, as we explained in the introduction [13]. Thus, this assumption simplifies the Good pH probe’s use and makes it accessible to more applications.

For the calculation of the pH value with the Good pH probe (${\mathrm{pH}}_{\mathrm{Good\,pH\,probe}}$), we combined Eq. (6) and Eq. (8):

9 $${\mathrm{pH}}_{\mathrm{Good\,pH\,probe}}=\mathrm{p}{K}_{\mathrm{a},\mathrm{MOPS}}+\mathrm{log}\left(\frac{{c}_{{\mathrm{MOPS}}^{-}}}{{c}_{\mathrm{MOPSH}}}\right).$$

To enable the application of the Good pH probe at different temperatures, $\mathrm{p}{K}_{\mathrm{a},\mathrm{MOPS}}$ was calculated as a function of the temperature $T$ according to the empirical equation of Roy et al. [35]:

10 $$\mathrm{p}{K}_{\mathrm{a},\mathrm{MOPS}}=\frac{814.077 {\mathrm{K}}^{-1}}{T}+9.865-0.9501\cdot \mathrm{ln}\left(T\right).$$

The concentration ratio between ${c}_{{\mathrm{MOPS}}^{-}}$ and ${c}_{\mathrm{MOPSH}}$ was determined by Raman measurements in combination with IHM for the spectral evaluation.

All experiments on the calibration, characterization, and application of the Good pH probe were evaluated on the basis of the root mean square error (RMSE)

11 $$\mathrm{RMSE}=\sqrt{\frac{1}{n}\sum_{k=1}^{n}{\left({\mathrm{pH}}_{\mathrm{Good\,pH\,probe},k}-{\mathrm{pH}}_{\mathrm{pH\,electrode},k}\right)}^{2} },$$

where ${\mathrm{pH}}_{\mathrm{pH\,electrode}}$ is the pH value measured with the reference pH electrode, $n$ is the number of data points, and $k$ is the running index. To be more precise, we evaluated the Good pH probe’s calibration with the RMSE of cross-validation (RMSECV). The RMSECV was calculated using a leave-one-out cross-validation approach, in which we determined the ${\mathrm{pH}}_{\mathrm{Good\,pH\,probe}}$ for each data point in the calibration dataset, while we used the remaining dataset for the calibration of the Good pH probe [34, 36]. To evaluate the characterization and application of the Good pH probe, we determined the RMSE of prediction (RMSEP). Both the RMSECV and the RMSEP were calculated according to Eq. (11).

### Spectral evaluation using IHM

In the IHM method, a mixture spectrum is interpreted as the weighted sum of the respective pure component spectra [33]. The results of a spectral evaluation using IHM are the IHM model weights ${\zeta }_{i}$, which correlate linearly with the corresponding concentrations ${c}_{i}$:

12 $${\zeta }_{i}\propto {c}_{i}.$$

For the application of IHM, a pure component spectrum is modeled as a sum of pseudo-Voigt functions, which are defined by four peak parameters: height, position, linewidth, and Gaussian fraction. These parameterized peak functions allow us to consider nonlinear mixture effects such as peak broadening or peak shifts by implementing specific peak parameters as degrees of freedom in the model.

**Fig. 2:**
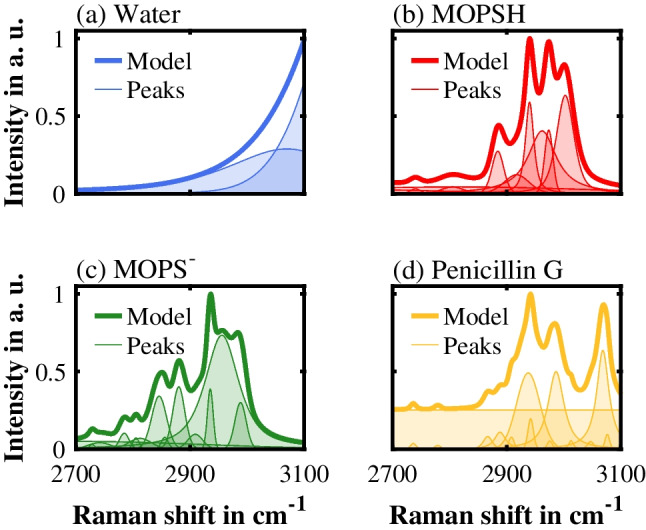
The pure component models of water (**a**), protonated MOPS ($\mathrm{MOPSH}$) (**b**), deprotonated MOPS (${\mathrm{MOPS}}^{-}$) (**c**), and penicillin G (**d**)

**Fig. 3:**
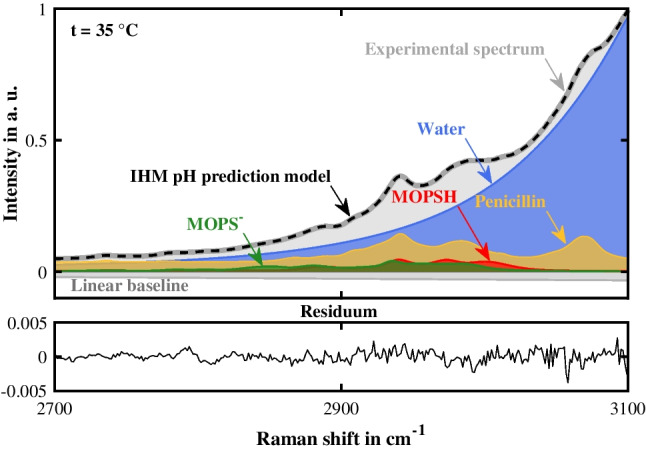
Exemplary fit of the Indirect Hard Modeling (IHM) pH prediction model (black dashed line) to an experimental Raman spectrum (dark gray solid line) of an aqueous solution containing 50 mM MOPS and 50 mM penicillin G, at 35 °C and pH 6.99. The spectrum that is modeled by the IHM pH prediction model is the sum of the pure component models and the linear baseline. The residuum is the difference between the experimental and the modeled spectrum. To improve clarity, the peak functions of the pure component models are not shown

To apply IHM in this study, an in-house MATLAB script was used. We developed an IHM pH prediction model for the evaluation of Raman spectra in the spectral range from 2700 to 3100 cm^−1^. This IHM pH prediction model consisted of a pure component model for water (2 peaks), a pure component model for $\mathrm{MOPSH}$ (10 peaks), and a pure component model for ${\mathrm{MOPS}}^{-}$ (14 peaks). The pure component models of water, $\mathrm{MOPSH}$, and ${\mathrm{MOPS}}^{-}$ including all peak functions are shown in Fig. 2a, b, and c. Since the Raman signal of water changes its shape as a function of the temperature and the ionic strength, we implemented the position and the linewidth of both water peaks as degrees of freedom in the IHM pH prediction model. For evaluating Raman spectra of penicillin G-containing samples, the IHM pH prediction model was extended with a pure component model for penicillin G (14 peaks) (Fig. 2d). Furthermore, the IHM pH prediction model included a linear baseline. An exemplary fit of the IHM pH prediction model to a Raman spectrum of an aqueous solution at 35 °C and pH 6.99 containing 50 mM MOPS and 50 mM penicillin G is shown in Fig. 3.

To develop these four pure component models, their respective pure component spectra were required. All spectra used for the development of the IHM pH prediction model were recorded at 35 °C. Although the IHM pH prediction model was developed at a specific temperature (35 °C), its application at other temperatures did not require any additional adjustments. Unlike water, $\mathrm{MOPSH}$, ${\mathrm{MOPS}}^{-}$, and penicillin G are no liquids in their pure form. Therefore, for these three components, spectra of aqueous 50 mM MOPS or 50 mM penicillin G solutions were used in combination with Complemental Hard Modeling (CHM) [37]. CHM allowed us to subtract the water model from these binary Raman spectra during the development of the respective pure component models. To record aqueous Raman spectra of almost completely protonated or deprotonated MOPS, we adjusted the pH value to either pH 2 or pH 12 by adding HCl or NaOH solution.

For the calibration of the IHM pH prediction model, we correlated the concentration ratio between ${c}_{{\mathrm{MOPS}}^{-}}$ and ${c}_{\mathrm{MOPSH}}$ with the IHM model weight ratio between ${\zeta }_{{\mathrm{MOPS}}^{-}}$ and ${\zeta }_{\mathrm{MOPSH}}$ via the calibration factor $k$:

13 $$\frac{{c}_{{\mathrm{MOPS}}^{-}}}{{c}_{\mathrm{MOPSH}}}=k\frac{{\zeta }_{{\mathrm{MOPS}}^{-}}}{{\zeta }_{\mathrm{MOPSH}}}.$$

As the concentrations of the two MOPS species were not known by weighing, this concentration ratio was calculated from the reference pH value according to Eq. (9). A ratiometric calibration, as we used in this study, has the additional advantage that it compensates for effects such as a fluctuating laser power. The calibration factor $k$ enabled the determination of the concentration ratio between ${c}_{{\mathrm{MOPS}}^{-}}$ and ${c}_{\mathrm{MOPSH}}$ of an unknown sample via the IHM model weights ${\zeta }_{{\mathrm{MOPS}}^{-}}$ and ${\zeta }_{\mathrm{MOPSH}}$.

## Results and discussion

In this study, the Good pH probe was calibrated, characterized, and proven to be applicable for non-invasive pH in-line monitoring in bioprocesses during an enzyme-catalyzed reaction.

### Calibration of the Good pH probe

To calibrate the Good pH probe, three identical standard experiments (“Experimental procedure” section) were performed at 35 °C. In these experiments, the pH value of aqueous 50 mM MOPS solutions was gradually increased within the effective working range of MOPS (pH 6 to pH 8) by adding NaOH solution. In total, the calibration dataset of the Good pH probe consisted of 60 data points.

**Fig. 4:**
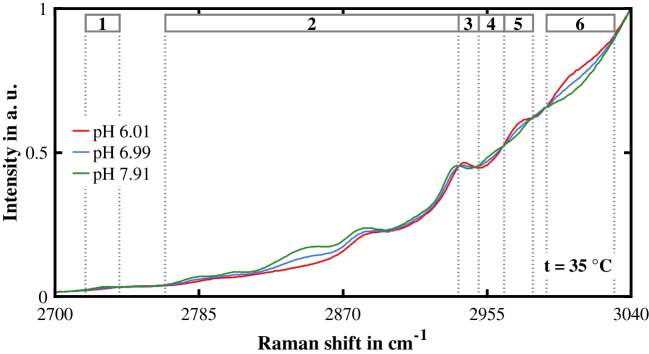
Exemplary calibration spectra of the Good pH probe. To emphasize the MOPS-specific peaks in the calibration spectra, the spectral range from 2700 to 3040 cm^−1^ is shown. The numbers 1 to 6 denote spectral ranges in which MOPS-specific peaks change as a function of the pH value. All spectra were normalized after a constant background was subtracted

Figure 4 presents three exemplary calibration spectra measured at pH 6.01, pH 6.99, and pH 7.91. Although the IHM pH prediction model was developed and applied in the spectral range from 2700 to 3100 cm^−1^, we visualized these calibration spectra in the spectral range from 2700 to 3040 cm^−1^ to highlight the MOPS-specific peaks. As the pH value rose, the Raman intensity of the MOPS-specific peaks increased in the spectral ranges from 2718 to 2738 cm^−1^, from 2765 to 2938 cm^−1^, and from 2950 to 2965 cm^−1^ (1, 2, and 4 in Fig. 4). Conversely, the Raman intensity of the MOPS-specific peaks decreased in the spectral ranges from 2938 to 2950 cm^−1^, from 2965 to 2982 cm^−1^, and from 2990 to 3030 cm^−1^ (3, 5, and 6 in Fig. 4). Changes in the MOPS-specific peaks were expected because MOPS deprotonates as the pH value rises, and thus the concentration of ${\mathrm{MOPS}}^{-}$ increases while the concentration of $\mathrm{MOPSH}$ decreases. Our IHM pH prediction model took these changes into consideration by superimposing the pure component models of ${\mathrm{MOPS}}^{-}$ and $\mathrm{MOPSH}$. In Table 1, the effect of the pH value on the MOPS-specific peaks is summarized.

**Table 1:** Effect of the pH value on the MOPS-specific peaks in the calibration spectra of the Good pH probe. This effect is defined as either "increase" or "decrease" depending on how the MOPS-specific peaks change with rising pH value

No.	Spectral range in cm^−1^	Effect
1	2718 to 2738	Increase
2	2765 to 2938	Increase
3	2938 to 2950	Decrease
4	2950 to 2965	Increase
5	2965 to 2982	Decrease
6	2990 to 3030	Decrease

**Fig. 5:**
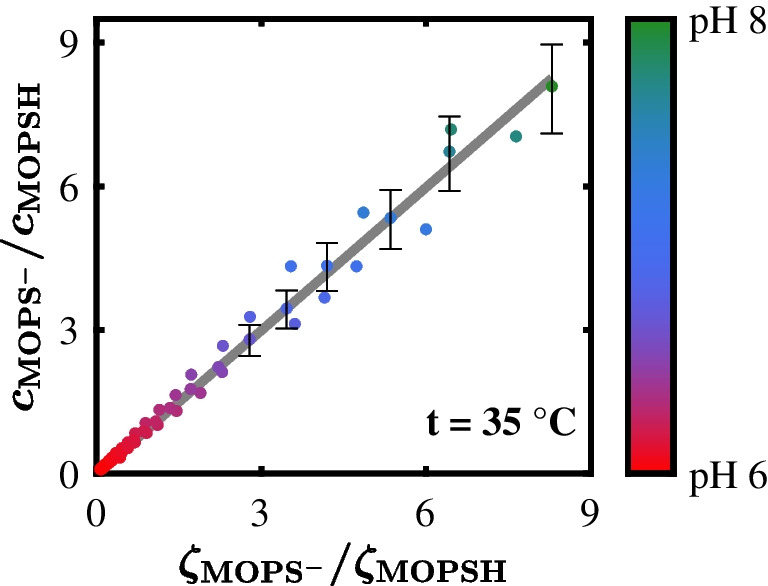
Calibration curve of the Good pH probe. The ratio between the concentration of deprotonated ${c}_{{\mathrm{MOPS}}^{-}}$ and protonated MOPS ${c}_{\mathrm{MOPSH}}$ is plotted against the corresponding ratio between the Indirect Hard Modeling (IHM) model weights of deprotonated ${\zeta }_{{\mathrm{MOPS}}^{-}}$ and protonated MOPS ${\zeta }_{\mathrm{MOPSH}}$. The ratio between ${c}_{{\mathrm{MOPS}}^{-}}$ and ${c}_{\mathrm{MOPSH}}$ was calculated from the reference pH value

The calibration curve of the Good pH probe, described by Eq. (13), is depicted in Fig. 5. This calibration curve allowed us to determine the calibration factor, which was 0.996. The proximity of this calibration factor to 1 matched our expectations, since the calibration factor of a ratiometrically calibrated IHM model [Eq. (13)] is basically the ratio of the respective component’s scattering cross sections, which in turn are supposed to be similar if the molecular structures of these components are similar [28, 38].

The calibration data showed a strong linear correlation (Fig. 5). This conclusion is supported by the RMSECV of 0.05 pH levels, which was within the range of the reference pH electrode’s measurement error. However, the fluctuation of the calibration data points increased with higher pH values. This increase in fluctuation was due to the calculation of the concentration ratio between ${c}_{{\mathrm{MOPS}}^{-}}$ and ${c}_{\mathrm{MOPSH}}$. As this ratio was calculated according to Eq. (9) from the reference pH value, the measurement error of the reference pH electrode led to an exponential increase in the fluctuation, indicated by exemplary error bars in Fig. 5.

### Characterization of the Good pH probe

The characterization of the Good pH probe included four aspects.

#### Reversibility

First, we investigated the reversibility of the Good pH probe. The reversibility of a measurement technique describes its ability to provide the same measured value for a certain process state regardless of the previous condition of the system. Therefore, an extended standard experiment (“Experimental procedure” section) at 35 °C was performed: after increasing the pH value of an aqueous 50 mM MOPS solution from pH 6 to pH 8 by adding NaOH solution, the pH value was decreased again to pH 6 by the addition of HCl solution.

**Fig. 6:**
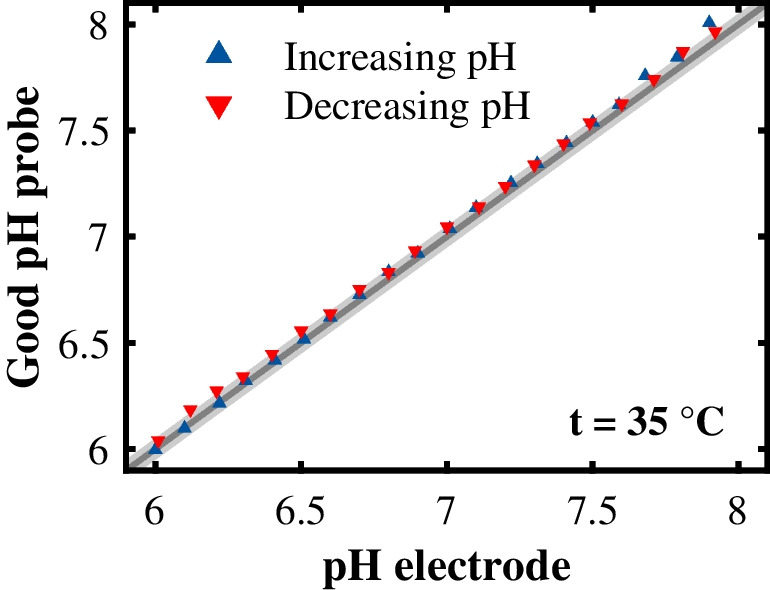
Reversibility of the Good pH probe. The prediction quality of the Good pH probe was evaluated for both increasing (NaOH) and decreasing (HCl) pH values. The pH prediction of the Good pH probe is plotted against the pH value measured with the reference pH electrode. The area in gray highlights the measurement error of the reference pH electrode

Figure 6 shows that the Good pH probe’s pH prediction for this extended standard experiment was not affected by the previous condition of the system, in this case the previous pH value. The RMSEP for increasing the pH value was 0.04 pH levels and the RMSEP for decreasing the pH value was 0.05 pH levels. These RMSEPs were within the measurement error of the reference pH electrode. Consequently, the Good pH probe proved to be reversible.

#### Temperature dependence

Second, we assessed whether the pH prediction of the Good pH probe showed temperature dependence. The temperature is one of the main influencing factors and a source of inaccuracy for spectroscopic pH monitoring, since this kind of pH monitoring is based on pH indicators that protonate or deprotonate as a function of the pH value. Besides the pH value, the temperature also influences the pH indicator’s state of protonation, as the acid dissociation constant is temperature-dependent. We investigated the temperature dependence of the Good pH probe in the temperature range between 15 and 40 °C, which is the relevant range for the majority of bioprocesses. For this purpose, we performed additional standard experiments (“Experimental procedure” section) at 15, 20, 25, 30, 35, and 40 °C, in which the pH value of aqueous 50 mM MOPS solutions was increased from pH 6 to pH 8 by the addition of NaOH solution.

**Fig. 7:**
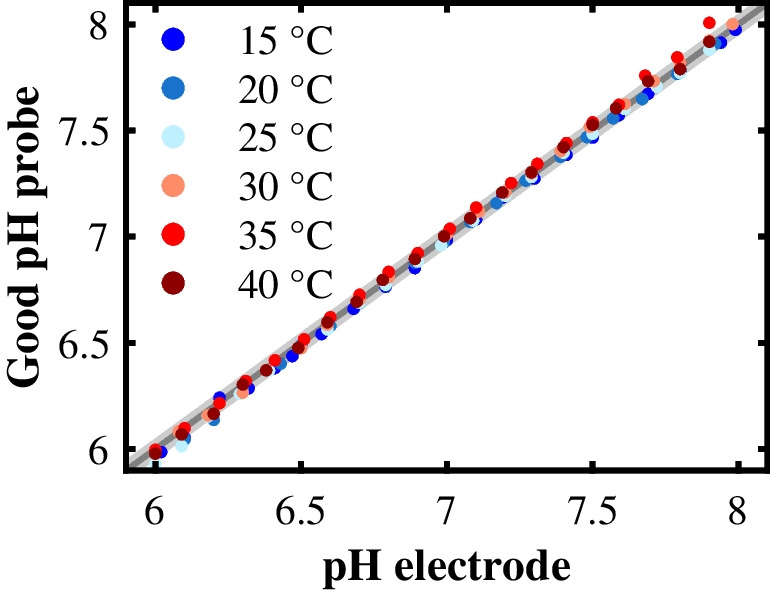
Temperature dependence of the Good pH probe. The pH prediction of the Good pH probe at six different temperatures between 15 and 40 °C is plotted against the pH value measured with the reference pH electrode. The area in gray highlights the measurement error of the reference pH electrode

Figure 7 shows the prediction quality of the Good pH probe at these temperatures. The RMSEPs of the Good pH probe at 15 °C (0.03 pH levels), 20 °C (0.02 pH levels), 25 °C (0.02 pH levels), 30 °C (0.03 pH levels), 35 °C (0.04 pH levels), and 40 °C (0.04 pH levels) were all within the range of the reference pH electrode’s measurement error, and Fig. 7 shows no systematic deviations in the prediction quality depending on the temperature. However, we must emphasize that the Good pH probe requires the temperature associated with each measurement point, since the calculation of the pH value according to Eqs. (9) and (10) involves the negative common logarithm of MOPS’s acid dissociation constant $\mathrm{p}{K}_{\mathrm{a},\mathrm{MOPS}}$ as a function of temperature. As the temperature is known for most bioprocesses, this requirement will not limit the scope of application for the Good pH probe.

#### Sensitivity to the ionic strength

Third, we investigated the sensitivity of the Good pH probe to the ionic strength. As stated in the introduction, the ionic strength is a common challenge for spectroscopic pH monitoring, which potentially limits its accuracy if activity coefficients are not estimated. For the Good pH probe, we assumed in the “Calculation of the pH value” section that the common logarithm of the ratio between ${\gamma }_{{\mathrm{MOPS}}^{-}}$ and ${\gamma }_{\mathrm{MOPSH}}$ was equal to 0 [Eq. (8)] and thus eliminated the need to estimate activity coefficients for the Good pH probe.

To classify to what extent this assumption influences the prediction quality of the Good pH probe, we conducted three standard experiments (“Experimental procedure” section) at 35 °C, in which we gradually increased the ionic strength of aqueous 50 mM MOPS solutions to approximately 1100 mM by the addition of NaCl. These three experiments differed only in their initial pH value, which we set to either pH 6.3, pH 7.0, or pH 7.7 by adding NaOH solution to cover the effective working range of the Good pH probe. Each of these experiments comprised 31 NaCl additions. The first 12 NaCl additions were five times smaller than the subsequent 19 NaCl additions because we wanted to more closely examine the range of ionic strengths in which the other experiments of this study were performed.

**Fig. 8:**
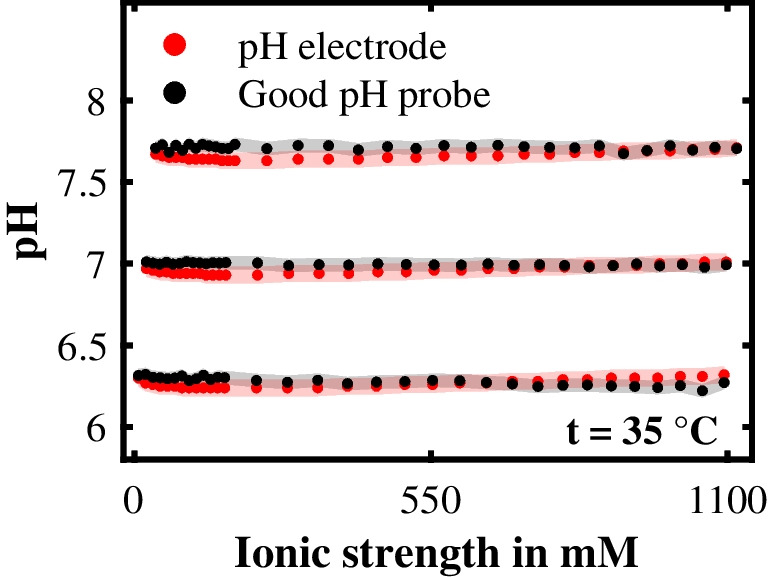
The Good pH probe’s sensitivity to the ionic strength. For three experiments, each differing in preset pH value (pH 6.3, pH 7.0, pH 7.7), the ionic strength was increased to approximately 1100 mM by the addition of NaCl. The pH prediction of the Good pH probe and the pH value measured with the reference pH electrode are plotted against the ionic strength in mM for these three experiments. The area in gray indicates the Good pH probe’s measurement error, and the area in light red highlights the reference pH electrode’s measurement error

In Fig. 8, the pH prediction of the Good pH probe at these different ionic strengths is compared with the measurement data of the reference pH electrode. Besides the Good pH probe’s pH prediction and the reference pH value, Fig. 8 also shows the measurement error of the Good pH probe, defined as the RMSEP at 35 °C, and the measurement error of the reference pH electrode. Figure 8 demonstrates that the Good pH probe’s pH predictions are either within the reference error or that the probe’s error at least overlaps with the reference error, indicating an accurate pH prediction over a wide range of ionic strengths. This observation was further supported by the corresponding RMSEPs of the Good pH probe at pH 6.3, pH 7.0, and pH 7.7, which were 0.05 pH levels, 0.05 pH levels, and 0.06 pH levels, respectively. All RMSEPs were within the measurement error of the reference pH electrode, except for the RMSEP at pH 7.7, which only slightly exceeded this error. These results indicated low sensitivity of the Good pH probe to the ionic strength and therefore supported the assumption we made in Eq. (8).

#### Applicability in more complex systems

Fourth, we investigated the applicability of the Good pH probe in systems where the spectral features of MOPS are strongly superimposed by additional components. Spectral superimposition is a scenario commonly encountered in the Good pH probe’s intended scope of application: non-invasive pH in-line monitoring in bioprocesses. We, therefore, added penicillin G, which strongly superimposes the spectral features of MOPS (Fig. 3) and is a commonly used component in bioprocesses, to the aqueous 50 mM MOPS solution that was used so far for calibration and characterization. We conducted three standard experiments (“Experimental procedure” section) at 35 °C, in which we increased the pH from pH 6 to pH 8 in aqueous solutions containing 50 mM MOPS and 50 mM penicillin G by the addition of NaOH solution.

**Fig. 9:**
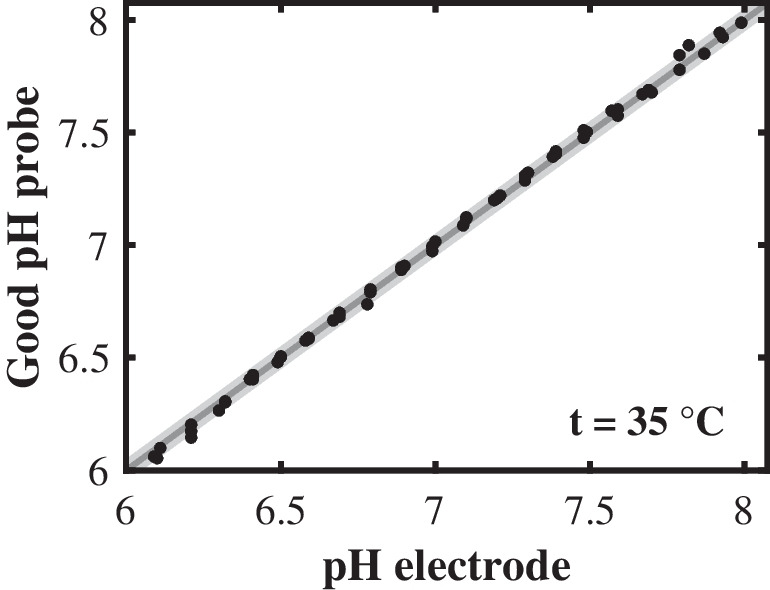
Applicability of the Good pH probe in a more complex system in which penicillin G significantly superimposes the spectral features of MOPS. The pH prediction of the Good pH probe is plotted against the pH value measured with the reference pH electrode. The area in gray highlights the measurement error of the reference pH electrode

Figure 9 illustrates the prediction quality of the Good pH probe in these solutions. For the spectral evaluation of this system, the IHM pH prediction model of the Good pH probe was extended with a pure component model for penicillin G. The RMSEP of all three penicillin G-containing experiments was 0.02 pH levels. Consequently, the Good pH probe is also applicable in more complex systems with significant spectral superimposition.

The prediction quality of the Good pH probe was not degraded by the additional spectral superimposing component, since it uses the physics-based IHM for spectral evaluation. A calibrated IHM prediction model can be transferred to a more complex system by extending the model with the respective pure component models [34]. This transfer does not affect the recently determined calibration factors as long as potential newly occurring nonlinear mixture effects are covered by additional adjustable peak parameters in the model. Transferability by extendibility is a feature of IHM and makes the Good pH probe extremely versatile.

During the characterization, we demonstrated that the Good pH probe is reversible, does not show any temperature dependence between 15 and 40 °C, exhibits low sensitivity to the ionic strength up to 1100 mM, and is applicable in more complex systems despite significant spectral superimposition. All RMSEPs of the characterization are summarized in Table 2.

**Table 2:** Characterization of the Good pH probe

Characteristic	RMSEP in pH levels
Reversibility	0.04 (pH increase), 0.05 (pH decrease)
Temperature dependence	0.03 (15 °C), 0.02 (20 °C), 0.02 (25 °C), 0.03 (30 °C), 0.04 (35 °C), 0.04 (40 °C)
Influence of ionic strength	0.05 (pH 6.3), 0.05 (pH 7.0), 0.06 (pH 7.7)
Applicability in case of spectral superimposition	0.03 (experiment 1), 0.02 (experiment 2), 0.02 (experiment 3)

### pH in-line monitoring during an enzyme-catalyzed reaction

**Fig. 10:**
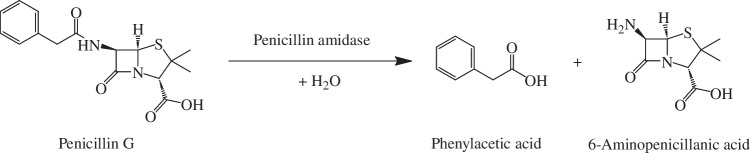
Hydrolysis of penicillin G to 6-aminopenicillanic acid and phenylacetic acid by penicillin amidase

To prove the applicability of the Good pH probe in bioprocesses, we used the probe for non-invasive pH in-line monitoring during an industrially relevant enzyme-catalyzed reaction [3]. This reaction was the hydrolysis of penicillin G to 6-aminopenicillanic acid and phenylacetic acid catalyzed by penicillin amidase (Fig. 10). During this reaction, the pH value decreases, since for each molecule of penicillin G that reacts, two new acid molecules, 6-aminopenicillanic acid and phenylacetic acid, are formed. As illustrated in Fig. 10, the carboxyl group of 6-aminopenicillanic acid is the former carboxyl group of penicillin G. Since penicillin G is a strong acid, it is deprotonated between pH 6 and pH 8, which corresponds to the effective working range of the Good pH probe [39]. Therefore, the carboxyl group of 6-aminopenicillanic acid was already deprotonated and did not affect the pH value. However, the carboxyl group of phenylacetic acid was newly formed during the enzyme-catalyzed reaction and led to a decrease in the pH value.

**Fig. 11:**
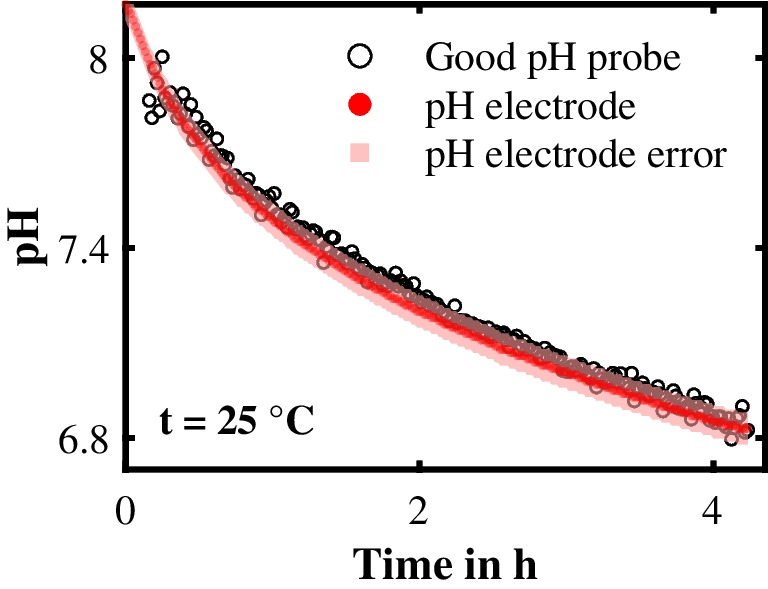
Non-invasive pH in-line monitoring with the Good pH probe during the hydrolysis of penicillin G to 6-aminopenicillanic acid and phenylacetic acid catalyzed by penicillin amidase. The pH prediction of the Good pH probe and the pH value measured with the reference pH electrode are plotted against the time in hours. The area in light red indicates the measurement error of the reference pH electrode

The pH in-line monitoring results for the Good pH probe are shown in Fig. 11. As soon as the pH value decreased to pH 8, the Good pH probe’s pH monitoring started, and after 4.23 h, the final pH value was pH 6.83. The Good pH probe accurately predicted the trend in the pH value during the enzyme-catalyzed reaction, and no systematic deviations were observed within the measurement error of the reference pH electrode. This conclusion is supported by the RMSEP of 0.04 pH levels.

The response time for the Good pH probe during the in-line monitoring of this enzyme-catalyzed reaction was approximately 68 s. This response time is comparable to that of commercial pH electrodes [40]. As the response time of the Good pH probe was mainly subject to the excitation time of one accumulated Raman spectrum (“Raman spectra” section), it can be further improved by shortening the excitation time. Finally, we proved the applicability of the Good pH probe for non-invasive pH in-line monitoring in an enzyme-catalyzed reaction.

### Evaluation of the Good pH probe

Using the Good pH probe for spectroscopic pH in-line monitoring in bioprocesses offers many advantages: As described in the introduction, it is completely non-invasive, since it utilizes the Good buffer MOPS, a pH buffer that is already part of many bioprocesses, as pH indicator. The Good pH probe does not require the estimation of activity coefficients, because MOPS exhibits the lowest possible charge for a pH indicator, as we explained in the “Calculation of the pH value” section and validated in the “Characterization of the Good pH probe” section. Moreover, the Good pH probe is reversible and temperature-independent between 15 and 40 °C (“Characterization of the Good pH probe” section). Although it was developed on a scale of several milliliters, it will also be applicable in small-scale systems such as microtiter plates or microchannels.

Despite all these advantages, the successful application of the Good pH probe is confronted with challenges. The evaluation of bioprocess Raman spectra is complex: While the IHM pH prediction model does not require recalibration for each new bioprocess to be monitored, because of transferability by extendibility (“Characterization of the Good pH probe” section), it does need to be extended and updated. Moreover, the Good pH probe is only applicable to bioprocesses that use a Good buffer to stabilize the process’s pH value. Lastly, as Good buffers are designed to effectively buffer in the physiological pH range, the effective working range of the Good pH probe is limited to approximately two pH levels in this pH range.

Commercially available systems for spectroscopic pH monitoring such as those from Ocean Insight or PreSens Precision Sensing combine pH indicators immobilized in sensor spots with absorption or fluorescence spectroscopy [6, 13]. Compared with the Good pH probe, these systems offer a wider effective working range, while their performance in terms of accuracy and response time is similar. However, unlike the Good pH probe, which uses Raman spectroscopy, these approaches cannot simultaneously monitor additional critical process parameters (e.g., substrate, metabolite, and product concentrations), nor are they completely non-invasive.

Olaetxea et al. [21], Metcalfe et al. [22], and Schenk et al. [20] have contributed to spectroscopic pH monitoring in bioprocesses, each advocating completely non-invasive approaches. For instance, Olaetxea et al. [21] used hemoglobin as pH indicator in combination with Raman spectroscopy to monitor the pH value in ex vivo pig blood samples, which demonstrated the potential of their approach for in vivo applications. Contrary to Olaetxea et al. [21], we do not intend to apply the Good pH probe in vivo, since Good buffers are not already part of living organisms and are designed to have low biological membrane permeability. Metcalfe et al. [22] and Schenk et al. [20] both used phosphate buffer as pH indicator and combined it with Raman or mid-infrared spectroscopy to on-line monitor the pH value during *E. coli* fermentations. Their effective working range and the prediction quality are comparable to the Good pH probe. Unlike in our study, however, in these two studies, the estimation of the activity coefficients was required.

What remains to be shown is whether the Good pH probe can be successfully applied to more complex bioprocesses beyond enzyme reactions, such as *E. coli* fermentations. In this regard, our previous study has already demonstrated that the combination of Raman spectroscopy and IHM is capable of monitoring other critical process parameters in glucose to ethanol fermentations by *Saccharomyces cerevisiae* (*S. cerevisiae*) [34].

## Conclusion

In this study, we successfully developed the Good pH probe, which is applicable in the effective working range from pH 6 to pH 8 and does not require the estimation of activity coefficients. The Good pH probe uses the Good buffer MOPS as pH indicator, Raman spectroscopy as spectroscopic technique, and IHM for the spectral evaluation. As MOPS is a frequently used pH buffer in bioprocesses, the Good pH probe is completely non-invasive.

To calibrate the Good pH probe, we used three calibration experiments, each comprising 20 Raman spectra. During characterization, we were able to prove that the Good pH probe is reversible, shows no temperature dependence in the biological relevant temperature range between 15 and 40 °C, exhibits low sensitivity to the ionic strength up to approximately 1100 mM, and is applicable in systems that show massive spectral superimposition with the spectral features of MOPS. For all characterization experiments, the pH prediction quality of the Good pH probe was within the expected error of the reference pH electrode (± 0.05 pH levels). Finally, we successfully applied the Good pH probe for the non-invasive pH in-line monitoring of an industrially relevant enzyme-catalyzed reaction. This enzyme-catalyzed reaction was the hydrolysis of penicillin G to 6-aminopenicillanic acid and phenylacetic acid by penicillin amidase, in which the pH value continuously decreased. In this enzyme-catalyzed reaction, we obtained an RMSEP of 0.04 pH levels, which was again within the expected error of the reference pH electrode.

The objectives of future studies will be to combine the Good pH probe with further Good buffers, to use the probe for non-invasive pH in-line monitoring of other bioprocesses (e.g., fermentations or cell cultures), and to apply the Good pH probe in small-scale systems, which may include microtiter plates or microchannels.

To conclude, we proved the Good pH probe to be an effective approach for non-invasive pH in-line monitoring, offering accurate measurements and applicability in bioprocesses, while demonstrating robustness against influencing factors such as temperature and ionic strength. Furthermore, with the Good pH probe, we added the pH value to the list of critical process parameters that can be monitored using Raman spectroscopy and IHM.

## Data Availability

The data that support the findings of this study are available from the corresponding author upon reasonable request.
